# Host transcriptional response to TB preventive therapy differentiates two sub-groups of IGRA-positive individuals

**DOI:** 10.1016/j.tube.2020.102033

**Published:** 2021-03

**Authors:** Claire Broderick, Jacqueline M. Cliff, Ji-Sook Lee, Myrsini Kaforou, David AJ. Moore

**Affiliations:** a Clinical Research Department, Faculty of Infectious and Tropical Diseases, London School of Hygiene and Tropical Medicine, Keppel St, London, WC1E 7HT, UK; b TB Centre, London School of Hygiene and Tropical Medicine, Keppel St, London, WC1E 7HT, UK; c Section for Paediatric Infectious Disease, Department of Infectious Disease, Faculty of Medicine, Imperial College London, London, W2 1PG, UK; d Department of Infection Biology, Faculty of Infectious and Tropical Diseases, London School of Hygiene and Tropical Medicine, Keppel St, London, WC1E 7HT, UK

**Keywords:** Latent tuberculosis infection, Preventive therapy, Transcriptome

## Abstract

We hypothesised that individuals with immunological sensitisation to *Mycobacterium tuberculosis* (*Mtb*), conventionally regarded as evidence of latent tuberculosis infection (LTBI), would demonstrate binary responses to preventive therapy (PT), reflecting the differential immunological consequences of the sterilisation of viable infection in those with active *Mtb* infection versus no *Mtb* killing in those who did not harbour viable bacilli.

We investigated longitudinal whole blood transcriptional profile responses to PT of Interferon gamma release assay (IGRA)-positive tuberculosis contacts and IGRA-negative, tuberculosis-unexposed controls. Longitudinal unsupervised clustering analysis with a subset of 474 most variable genes in antigen-stimulated blood separated the IGRA-positive participants into two distinct subgroups, one of which clustered with the IGRA-negative controls. 117 probes were differentially expressed over time between the two cluster groups, many of them associated with immunological pathways important in mycobacterial control.

We contend that the differential host RNA response reflects lack of *Mtb* viability in the group that clustered with the IGRA-negative unexposed controls, and *Mtb* viability in the group (1/3 of IGRA-positives) that clustered away.

Gene expression patterns in the blood of IGRA-positive individuals emerging during the course of PT, which reflect *Mtb* viability, could have major implications in the identification of risk of progression, treatment stratification and biomarker development.

## 1 Introduction

The term latent tuberculosis infection (LTBI) is loaded with the inference that viable *Mycobacterium tuberculosis (Mtb)* organisms are present in the affected individual which, under the right circumstances, have the capacity to cause reactivation and TB disease. Tests of immunological reactivity, whether delayed type hypersensitivity reactions measured in the tuberculin skin test (TST) or T lymphocyte stimulation though antigen recognition in the interferon gamma release assays (IGRAs) are widely referred to as tests for LTBI [1].

However, neither approach demonstrates presence of viable *Mtb* bacilli and there is no histopathological hallmark of LTBI. The lifetime risk of reactivation disease from a *Mtb* infection acquired remotely in time is around 10% [2]. In the interval between acquisition of infection and development of disease, *Mtb* maintains viability and is assumed to be slowly replicating, either under close immunological control or in a relatively immunologically privileged location. Thus, LTBI induces immunological sensitisation as reflected in the TST and IGRA, tests that demonstrate immunological memory for prior exposure to mycobacterial antigens.

Nevertheless, 90% of individuals demonstrating immunological recognition of *Mtb* antigens by positive IGRA or TST never develop active TB disease. Taking the inherent assumption that TST and IGRA are indicators of LTBI to its logical conclusion, the 90% who escape development of TB do so because the immune control-pathogen balance remains in favour of the human host. An alternative explanation might be that a large proportion of those with positive TST and IGRA testing do not harbour viable organisms and are thus incapable of progressing to reactivation TB.

Preventive therapy (PT), in which a limited course of anti-TB antibiotics is used to sterilise presumed viable infection in individuals with positive TST and/or IGRA tests, has been shown to be highly effective in reducing the risk of future TB disease [3].

We hypothesised that differentiation of LTBI with viable bacilli from immunological sensitisation without viable infection could be achieved by investigating the whole blood transcriptomic response to effective PT. We hypothesised that mycobacterial killing from effective LTBI PT would lead to a detectable alteration in the transcriptome that would not be seen in those individuals in whom there were no *Mtb* to be killed, whether these were IGRA/TST positive or healthy IGRA/TST negative controls with no known prior TB exposure.

## 2 Materials and methods

### 2.1 Ethics statement

The study procedures and protocol were approved by City & East NHS Research Ethics Committee, London (reference 16/LO/1206) and the London School of Hygiene and Tropical Medicine Research Ethics Committee (reference 11603). Written informed consent was given by all participants before inclusion in the study.

### 2.2 Participants

Study participants were recruited from National Health Service (NHS) tuberculosis (TB) outpatient clinics in London (Whittington Health NHS Trust, Royal Free London NHS Foundation Trust, Barts Health NHS Trust, Homerton University Hospital NHS Foundation Trust). Healthy controls were recruited from the London School of Hygiene and Tropical Medicine.

Participants were recruited who were aged 18 years and above, had positive Interferon gamma release assay (IGRA) (performed by the local hospital laboratories, using the QuantiFERON-TB Gold In-tube assay [Qiagen, Manchester, UK]), with known exposure to an index person with isoniazid- and rifampicin-susceptible pulmonary TB (contact history unconfirmed for three individuals) and who planned to initiate a 12-week course of combined rifampicin/isoniazid (RH) as preventive therapy (once daily rifampicin 600 mg/isoniazid 300 mg as Rifinah) plus once daily pyridoxine 10 mg. Adult volunteers aged 18 years and above were recruited as healthy control participants.

Once consented, demographic information, TB exposure history, and medical history were recorded on a data capture sheet and testing for human immunodeficiency virus (HIV) was performed. Healthy volunteers additionally underwent IGRA testing (performed using the QuantiFERON-TB Gold In-tube assay according to the manufacturer's recommendations) and were excluded if they were found to be IGRA+. Individuals were excluded if they had a prior history of TB infection, of having taken anti-TB treatment or exposure to drug-resistant TB. Participants who were pregnant, breastfeeding or trying to conceive, those with immunosuppressive disorders including HIV and those who had taken immunosuppressant medication in the preceding six months were also excluded. Healthy control participants reporting prior exposure to TB were also excluded.

Healthy controls were given a two-week course of RH (once daily rifampicin 600 mg/isoniazid 300 mg as Rifinah) plus once daily pyridoxine 10 mg.

Blood samples were collected from all participants at baseline (visit [V]1) and 2 weeks after initiating RH (V2), with an additional sample point in IGRA+ participants within 6 weeks of completion of the 12-week course of treatment (V3). At all sampling timepoints, all participants were asked about their adherence to treatment, and whole blood was collected in a PAXgene blood RNA tube (PreAnalytiX GmbH, Hombrechtikon, Switzerland) for RNA expression analysis and a lithium heparin tube (Becton Dickinson, Berkshire, UK) for subsequent stimulation assays. The PAXgene tubes were frozen within 4 h of collection.

### 2.3 Stimulation of whole blood

Stimulation was performed using QuantiFERON-TB Gold Plus In-tube Assay (QFT-TB Plus) (Qiagen). Within 4 h of collection, 1 ml of blood was transferred from the lithium heparin tube to each of the four QFT-TB Plus tubes: TB1 antigen, TB2 antigen (both containing peptides from ESAT-6 and CFP-10 antigens), mitogen positive control and (unstimulated) negative control. The tubes were gently shaken to dissolve the lyophilized peptides in the blood. The QFT-TB Plus tubes were immediately incubated upright at 37 °C for 22–24 h. After incubation, the blood was transferred into a 1.5 ml microcentrifuge tube and centrifuged for 15 min at 3000 RCF(g). Supernatants were removed and the remaining cell pellet (500 μl) was transferred into a 15 ml tube containing 2.5 ml RNAprotect® Cell Reagent (Qiagen). The cells were resuspended by vortexing, and incubated for 2 h for complete cell lysis before freezing at −80 °C.

### 2.4 Peripheral blood RNA expression by microarray

Total RNA was extracted from the PAXgene tubes using the PAXgene Blood miRNA Kit (Qiagen), and from the QFT-TB Plus stimulated samples, which had been lysed in RNAprotect, using the RNEasy mini kit (Qiagen), according to the manufacturer's instructions, incorporating on-column DNAse digestion. Globin depletion was performed using the GLOBINclear Kit (ThermoFisher), RNA was quantified by Nanodrop and the quality was assessed using an Agilent Bioanalyzer (Agilent, Cheshire, UK. The two-color low input Quick Amp Labelling kit (Agilent) was used to Cy3-or Cy5-fluorescently label cRNA samples, which were then hybridized to SurePrint G3 Human Gene Expression 60K GeneChip microarrays (Agilent) according to the manufacturer's instructions. Hybridization intensity was quantified via a SureScan Microarray Scanner (Agilent). Microarray data are deposited at Gene Expression Omnibus, Series **GSE153342**.

Individual channel intensities from the GeneChip data were extracted independently and analysed as separate observations [4].

### 2.5 Statistical analyses

Clinical data were analysed using ‘R’ Language and Environment for Statistical Computing 3.5.2. Fishers, Chi-squared and Kruskall Wallis tests of significance were used for categorical data. Mann-Whitney U tests of significance were used for continuous data.

Expression data were analysed using ‘R’ Language and Environment for Statistical Computing 3.5.2. Pre-processing, log-2 transformation and normalisation were performed using the Agilp package [5]. Microarrays were run using two batches of microarray slides and Principal Component Analysis identified an associated batch effect. Batch correction was performed using the COmBat function in the Surrogate Variable Analysis (sva) package in R [6,7]. To minimise the potential influence of batch correction on subsequent clustering analyses, no reference batch was used and independent COmBat-corrections were performed for each dataset of interest (individual PAXgene, TB1 and TB2 tube datasets and a combined TB1/TB2/negative tube dataset). Post-Combat correction PCA plots were undertaken to confirm the removal of the batch effect and identify outliers.

Differential gene expression analysis was performed using the limma package in R [8] which uses linear models. Where paired samples were available and analysis was relevant, paired t-tests were performed, with this being stated in the results. Adjustment for false discovery rate was performed using Benjamini-Hochberg (BH) correction with a significance level of adjusted p-value <0.05.

Prior to longitudinal analyses, the gene expression set was filtered to remove noise. Lowly expressed transcripts for which expression values did not exceed a value of 6 for any of the samples, were removed. Transcripts with extreme outlying values were removed, which were defined as values < (Quartile1 – [3* Inter-Quartile Range]) or > (Quartile3 + [3 * Inter-Quartile Range]). Transcripts with the greatest temporal and interpersonal variability were then selected based on their variance, with those transcripts with variance >0.1 taken forwards to the longitudinal analysis. X-chromosome transcripts which were significantly differentially expressed with gender at V1, V2 and/or V3 were identified using linear models in limma (BH corrected p value < 0.05) and were excluded, as were Y-chromosome transcripts.

Unsupervised longitudinal clustering analyses were performed using the BClustLong package in ‘R’ [9], which uses a Dirichlet process mixture model for clustering longitudinal gene expression data. A linear mixed-effects framework is used to model the trajectory of genes over time and it bases clustering on the regression coefficients obtained from all genes. 500 iterations were run (thinning by 2, so 1000 iterations in total).

Longitudinal differential gene expression analyses were performed using the MaSigPro package in R [10]. MaSigPro follows a two-step regression strategy to find genes with significant temporal expression changes and significant differences between groups. Coefficients obtained in the second regression model are then used to cluster together significant genes with similar expression patterns. Adjustment for false discovery rate was performed using BH correction with a significance level of adjusted p-value <0.05. Given the three timepoints from the IGRA+ individuals and the two timepoints from the healthy control groups, we employed both quadratic and linear approaches to account for all the potential curve shapes in the gene expression data.

Estimations of relative cellular abundances were calculated from the normalised full gene expression matrix (58,201 gene probes) using CibersortX [11], which uses gene expression data to deconvolve mixed cell populations. We used the LM22 [12] leukocyte gene signature matrix as reference, that comprises 22 different immune cell types, and ran 1000 permutations. Total monocyte fraction was calculated as the sum of the fractions of monocytes, macrophages and dendritic cells. Total lymphocyte fraction was calculated as the sum of B cells, Plasma cells, CD8^+^ T cells, CD4^+^ T cells, Helper follicular T cells, Regulatory T cells, Gamma delta T cells, and NK cells. A polynomial model (degrees of freedom = 2) was fitted in R to estimate relationships between the monocyte: lymphocyte ratio and time, in IGRA+ subgroups A and B.

## 3 Results

### 3.1 Recruitment of participants

**Figure 1:**
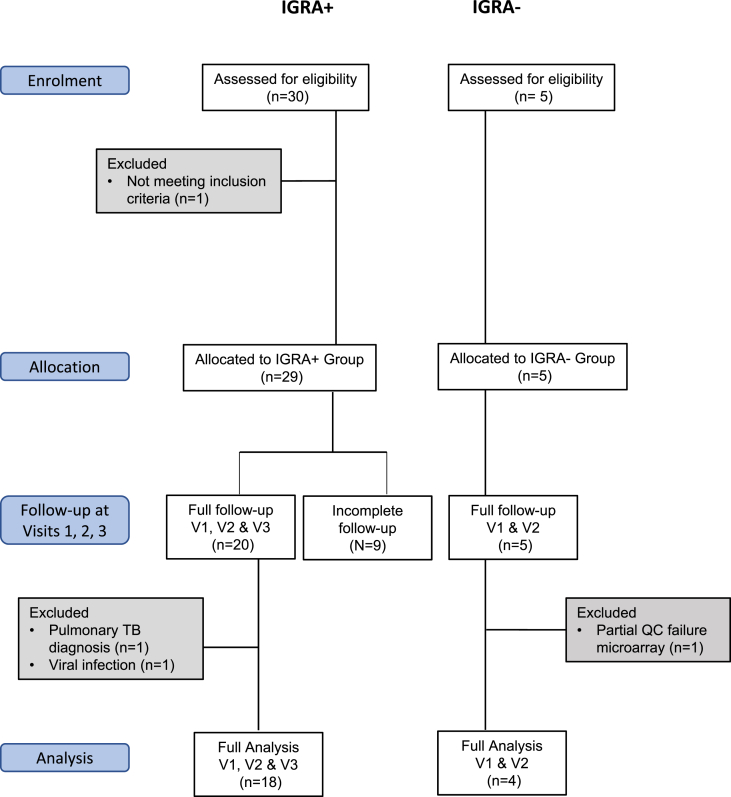
Study overview, showing patient numbers and exclusions.

Thirty adult IGRA-positive (IGRA+) participants were recruited to the study in the period October 2016 to January 2018, of whom 20 took a 12-week course of daily combined rifampicin/isoniazid (RH) as preventive therapy (PT) and completed study follow-up. Adult IGRA-negative (IGRA-) healthy volunteers were recruited to the study and completed a two-week course of daily RH. After quality control and pre-processing, 18 IGRA+ individuals and 4 IGRA- healthy controls were taken forward for comparator analyses (Fig. 1 and Supplementary Figs. 1 and 2). Recent exposure to drug-susceptible pulmonary TB was confirmed for 15/18 IGRA+s. There were no significant differences in age, gender, ethnicity or BCG status between the 18 IGRA+s and 4 IGRA- healthy controls (Table 1).

**Table 1:** Subject characteristics.

		IGRA+ group	IGRA- Healthy control group
Number		18	4
Age in years: Median (IQR)		34 (28–38)	28 (27–29)
Gender	Male	10 (56%)	3 (75%)
	Female	8 (44%)	1 (25%)
Confirmed recent drug-susceptible TB exposure	Yes	15 (83%)	0 (0%)
	No	3 (17%)	4 (100%)
BCG	Yes	14 (78%)	2 (50%)
	No	2 (11%)	2 (50%)
	Unknown	2 (11%)	0 (0%)
Continent of Birth	Africa	4 (22%)	0 (0%)
	Asia	4 (22%)	0 (0%)
	Australasia	0 (0%)	1 (25%)
	Europe	9 (50%)	2 (50%)
	North America	0 (0%)	1 (25%)
	South America	1 (6%)	0 (0%)
	Unknown	0 (0%)	0 (0%)
Ethnicity	Asian^a^	5 (28%)	2 (50%)
	Black^b^	4 (22%)	0 (0%)
	White^c^	8 (44%)	2 (50%)
	Other^d^	1 (6%)	0 (0%)

a Includes Bengali, Hong Kong, Kurdish, Sri Lankan, Turkish.

b Includes Black African.

c Includes White British, Polish, Romanian, White other.

d Includes Latin American, Unknown.

### 3.2 Comparing gene expression profiles for IGRA+ versus IGRA- participants

First, we evaluated whether there were discernable differences in gene expression between the IGRA+ participants and IGRA- healthy controls, using linear models [8]. In the unstimulated PAXgene blood samples, no transcripts were found to be significantly differentially expressed (SDE) between the IGRA+ and IGRA- participants at baseline (V1) or visit 2 (V2) (Benjamini-Hochberg [BH] corrected p value < 0.05).

**Figure 2:**
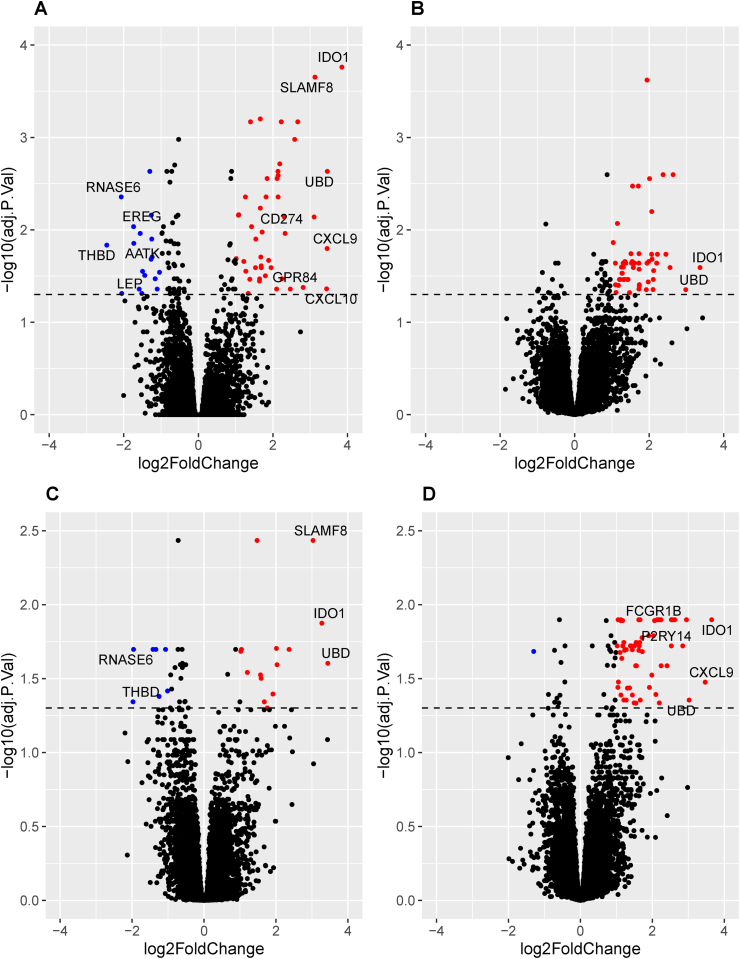
**Volcano plots showing genes significantly differentially expressed between IGRA+ and IGRA- individuals.** Plots are shown for TB1-stimulated samples at Visit (V) 1 [A] and V2 [B] and TB2-stimulated samples at V1 [C] and V2 [D]. Genes overexpressed in IGRA+s with log2Foldchange (LFC) > 1 and Benjamini-Hochberg adjusted p value ≤ 0.05 are shown in red. Genes underexpressed in IGRA+ individuals with LFC <-1 and BH adjusted p value ≤ 0.05 are shown in blue. Genes with LFC >2.7 and < −1.7 are annotated with their gene symbols. Dotted line denotes the significance cut-off (BH adjusted p value ≤ 0.05).

In this study, QuanitFERON-TB Gold Plus TB1 and TB2 tubes were used to stimulate whole blood. While both tubes contain peptides from ESAT-6 and CFP-10 *Mycobacterium tuberculosis* (*Mtb*) antigens, the TB1 tube peptides are designed to stimulate CD4^+^ T cells, and the TB2 peptides to stimulate both CD4^+^ and CD8^+^ T cells [13]. In contrast to the PAXgene tube whole blood samples, in the TB1-stimulated samples, 123 transcripts were SDE between IGRA+ and IGRA- individuals in the baseline (V1) samples and 93 were SDE between IGRA+ and IGRA- individuals in the V2 samples (BH corrected p value < 0.05) (Fig. 2A and B and listed in Supplementary File 1). In the TB2-stimulated blood samples, when IGRA+ individuals were compared to IGRA-, 43 transcripts were found to be SDE in the V1 samples and 86 in the V2 samples. (BH corrected p value < 0.05) (Fig. 2C and D and listed in Supplementary File 1). In summary, in vitro stimulation was necessary to distinguish the IGRA+ group from the IGRA- group.

### 3.3 Effects of stimulation on whole blood gene expression

In addition to the TB1 and TB2 *Mtb*-peptide-containing tubes, the QuantiFERON-TB Gold Plus kit also includes a “negative” tube which contains no mycobacterial antigen peptides We assessed the effects of stimulation by comparing gene expression in the TB1- and TB2- stimulated tubes versus the negative tube at visit 1, using paired t-tests. In the IGRA+ group, when TB1 tube samples were compared to the negative tube, 3578 transcripts were SDE, while 3217 transcripts were SDE in the TB2 tube samples versus the negative tube samples (BH corrected p value < 0.05), 2495 of which overlapped with the TB1 comparison (Supplementary Figs. 3A and 3B; SDE transcripts listed in Supplementary File 2). No genes were found to be SDE for the TB1- versus TB2-stimulated samples comparison.

In the IGRA- healthy controls, 37 transcripts were SDE in the TB1-stimulated samples compared to the negative tubes at visit 1 whereas just four transcripts were SDE in the TB2-stimulated samples (BH corrected p value < 0.05) (Supplementary Figs. 3C and 3D; SDE transcripts listed in Supplementary File 3).

### 3.4 Filtering the gene expression dataset

Analyses were focused on the stimulated samples, as there had been no detectable differences between the IGRA+ and IGRA- participants in the unstimulated PAXgene samples. As described above, stimulation induced changes in gene expression in the IGRA- healthy controls, with a higher number of SDE genes observed with TB1-stimulation than TB2- stimulation, suggesting a greater non-specific effect independent of *Mtb* infection in the TB1 stimulation. We were concerned these non-specific effects could provide interference, so focused on the TB2-stimulated samples for the next stage of the analysis.

The gene set was filtered to eliminate noise. Expression values of the 58,201 transcripts ranged from 4.4 to 18.7, so a conservative noise threshold of 6 was chosen. Of the remaining 34,110 transcripts, those with the greatest variability between participants and over time were selected for the analysis as described in 2.5. Through this process, a dataset with the “most variable genes” was generated for the TB2-stimulated samples (474 transcripts, listed in Supplementary File 4).

### 3.5 Clustering analysis of longitudinal gene expression

We hypothesised that the IGRA+ group is heterogeneous, containing individuals with viable mycobacteria who would demonstrate a transcriptomic response to PT, and IGRA+ individuals without viable mycobacteria, who would not demonstrate a transcriptomic response to PT and would more closely resemble the healthy control IGRA- group. To unmask the PT-specific transcriptomic responses, we sought to stratify the IGRA+ group of individuals in an agnostic way. We employed unsupervised clustering analysis of longitudinal gene expression in the 18 IGRA+ patients and the 4 IGRA- controls, aiming to identify IGRA+ subgroups, using the most variable 474 transcripts in the TB2-stimulated dataset. The BClustLong package in ‘R’ [14] was utilised, which uses a linear mixed-effects framework to model the trajectory of genes over time and bases clustering on the regression coefficients obtained from all genes.

This longitudinal clustering analysis revealed two subgroups of IGRA+ participants. One subgroup of IGRA+s (IGRA+ subgroup A, n = 12) clustered with the four healthy controls (Cluster 1), suggesting their gene expression over time was more similar to this *Mtb*-unexposed IGRA- population than it was to the remaining IGRA+s (IGRA+ subgroup B, n = 6) who formed Cluster 2. There were no significant differences in age, gender, ethnicity, BCG vaccination status or the IGRA+ participants’ TB contact history between Clusters 1 and 2 (Table 2).

**Table 2:** Characteristics of Cluster groups 1 and 2.

		BClustLong clustering group		
		Cluster 1	Cluster 2	p value
Number of participants		16	6	N/A
Patient IDs		HC51	LTBI6	N/A
		HC53	LTBI10	
		HC54	LTBI14	
		HC55	LTBI22	
		LTBI1	LTBI23	
		LTBI2	LTBI30	
		LTBI3		
		LTBI5		
		LTBI7		
		LTBI9		
		LTBI12		
		LTBI15		
		LTBI16		
		LTBI27		
		LTBI28		
Age in years: Median (IQR)		32.5 (24–41)	33.5 (29–38)	0.6
Gender	Male	9 (56%)	4 (66%)	1
	Female	7 (44%)	2 (33%)	
Confirmed recent exposure to DS-TB^a^	Yes	10 (83%)	5 (83%)	1
	No	2 (17%)	1 (17%)	
BCG	Yes	10 (62%)	6 (100%)	0.2
	No	4(25%)	0 (0%)	
	Unknown	2 (13%)	0 (0%)	
Continent of Birth	Africa	3 (19%)	1 (17%)	0.2
	Asia	1 (6%)	3 (50%)	
	Australasia	1 (6%)	0 (0%)	
	Europe	9 (56%)	2 (33%)	
	North America	1(6%)	0 (0%)	
	South America	1 (6%)	0 (0%)	
Ethnicity	Asian^b^	4 (25%)	3 (50%)	0.7
	Black^c^	3 (19%)	1 (17%)	
	White^d^	8 (50%)	2 (33%)	
	Other^e^	1 (6%)	0 (0%)	

a For IGRA+ participants only.

b Includes Bengali, Hong Kong, Kurdish, Sri Lankan, Turkish.

c Includes Black African.

d Includes White British, Polish, Romanian, White other.

e Includes Latin American, Unknown.

### 3.6 Longitudinal differential gene expression analysis

In order to unravel the underlying blood transcriptomic differences between the two cluster groups generated by the unsupervised clustering, we performed longitudinal differential gene expression analysis using MaSigPro package in R [10]. MaSigPro identifies genes with significant temporal expression changes and genes which are significantly differentially expressed between groups.

Of the 474 transcripts in the dataset, 117 transcripts, corresponding to 109 genes, were SDE over time between the two patient groups (with degrees of freedom = 1 capturing linear trends, BH corrected p value < 0.05, listed in Supplementary File 5), while 2 of these genes had significant linear terms associated with time (*P2RY6*, *SLC2A3*). Setting the degrees of freedom to 2, 69 out of the 117 genes were SDE over time between the two cluster groups (BH corrected p value < 0.05, listed in Supplementary File 5), while 4 of these genes (*MSR1*, *MT1CP*, *IGHG3*, *IGHG1*) also had significant linear and quadratic terms associated with time. In comparing Cluster 1 versus Cluster 2, when one of the clusters is heterogeneous (IGRA+ subgroup A plus IGRA- healthy controls), it is expected that some of the differences will be due to the IGRA+ subgroup B versus IGRA- comparison and not the IGRA+ subgroup B vs IGRA+ subgroup A comparison.

### 3.7 Biological relevance of the significantly differentially expressed genes

The biological relevance of the 117 transcripts significantly differentially expressed over time between the two patient cluster groups was investigated. Around one quarter of these SDE genes have been previously reported in transcriptomics studies comparing blood from TB patients with healthy controls (31 transcripts, 25 genes) or with other diseases (9 transcripts, 7 genes) [[15], [16], [17], [18], [19], [20], [21]]; (Supplementary File 5). Functional classification of these genes using PANTHER [22,23] revealed that 44/84 of the coding genes encode proteins with specific immunological functions, including cytokines, cytokine receptors and cytokine signaling (12), chemokines and chemokine-like proteins (11), immunoglobulins (9), immune cell receptors (4), antimicrobial peptides (3), complement (1) and antigen presentation (1) (Supplementary File 6).

**Figure 3:**
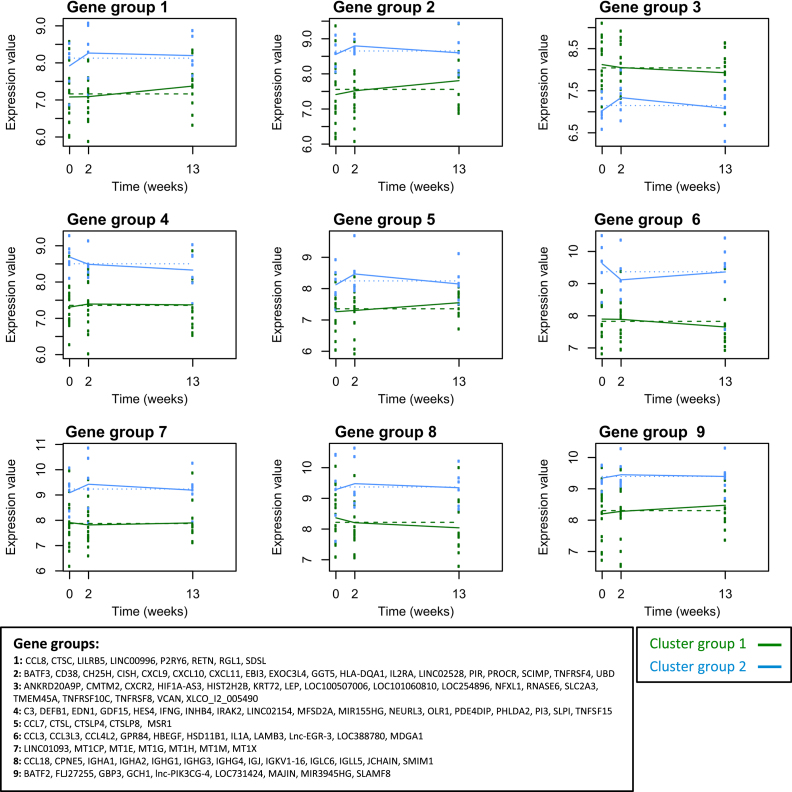
**Longitudinal differential gene expression analysis between patient cluster groups 1 and 2 in TB2-stimulated whole blood samples**. With 1° of freedom, 117/474 transcripts were SDE over time and between Cluster groups 1 and 2 (BH corrected p value < 0.05). The coefficients obtained were used to group together significant genes with similar longitudinal expression patterns. MaSigPro identified 9 gene groups. Plots of gene expression against time for these gene groups are shown for patient Cluster groups 1 (green) and 2 (blue). Lines join the median expression values of the gene groups at each timepoint. The gene symbols are listed for each gene group.

Coefficients obtained using MaSigPro were used to cluster significant genes with similar longitudinal expression patterns (Fig. 3). Often the proteins contained within a gene set had similar function, such as the CXC chemokines CXCL9, 10 and 11 in gene set 2 which were more highly expressed in patient Cluster 2 and increased at V2, and the pro-inflammatory NF-κB transcription factor-inducing proteins IFNγ, IL-1R associated kinase 2 (IRAK2) and TNF superfamily member 15 (TNFSF15) in gene set 4, which were more highly expressed in patient Cluster 2 and decreased through PT. BATF2, GCH1 and GBP3 all grouped in gene set 9, with consistently higher expression in patient Cluster 2. Gene expression was higher in patient Cluster 1 in only one gene set (gene set 3).

Biological pathways analysis was performed using Reactome pathway knowledgebase [24], with 80/117 transcripts successfully mapping to the database. Eleven pathways had significant over-representation of transcripts within our dataset (BH corrected p value < 0.05; listed in Supplementary file 7): these were all related to the immune system and encompassed pathways related to chemokine receptor binding, cytokine signaling – including IL10, TNF and regulatory T cells, metal ion binding and Complement cascade activation. There were a further 39 pathways with borderline over-representation: these largely encompassed biological functions related to innate immunity, antimicrobial peptides, phagocytosis, intracellular infection, and further cytokine signaling and Complement activation pathways.

### 3.8 Differing cellular responses to preventive therapy

**Fig. 4:**
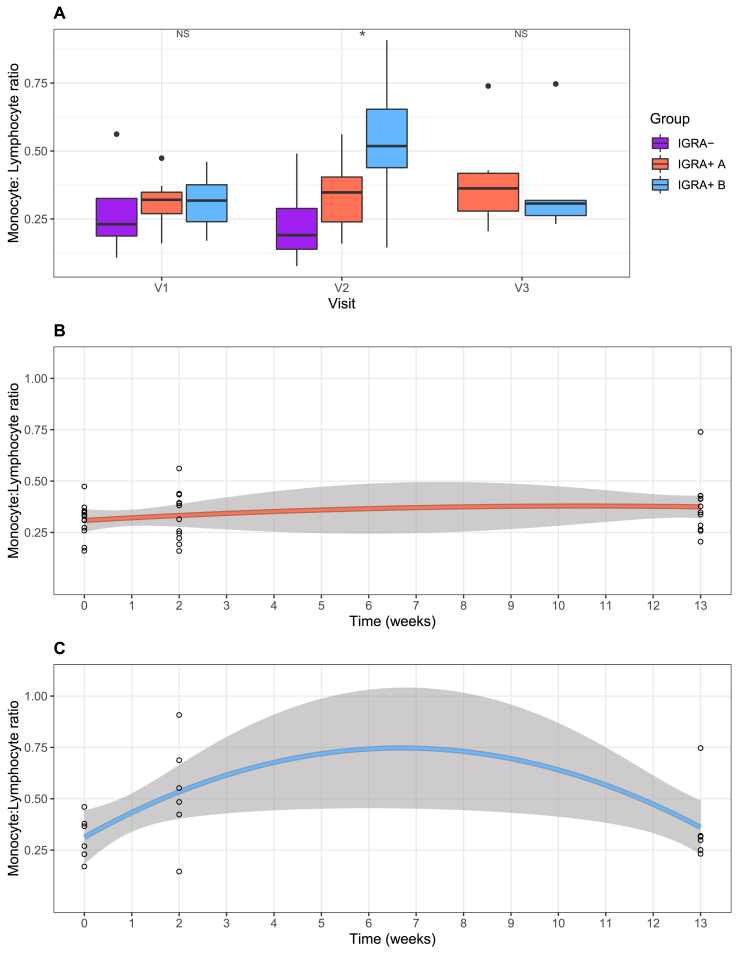
**Longitudinal changes in monocyte: lymphocyte ratio through preventive therapy in IGRA+ subgroups A and B.** Cibersortx was used to estimate the abundance of monocytes and lymphocytes in the TB2-stimulated whole blood samples at each visit, and the monocyte: lymphocyte ratio was calculated. (A) Boxplots showing the Monocyte: Lymphocyte ratios at Visits 1, 2 and 3 for IGRA- healthy controls and IGRA+groups A and B. NS denotes p > 0.05, * denotes p ≤ 0.05. Scatterplots showing the change in Monocyte: lymphocyte ratio over the time-course of the study period for (B) IGRA+subgroup A and (C) IGRA+subgroup B, where Visit 1 is 0 weeks, Visit 2 is 2 weeks and Visit 3 is 13 weeks, with 90% confidence intervals shown.

Relative cellular abundances were estimated from the gene expression data using CibersortX [11]. The estimated abundances of monocytes and lymphocytes were used to calculate the monocyte: lymphocyte ratio (MLR) for the two cluster groups at all three visits. At visits 1 and 3, the MLRs were similar between Clusters 1 and 2. However, at visit 2, they were higher in Cluster 2 (median = 0.52) compared to Cluster 1 (median = 0.29, p = 0.03). This difference at visit 2 remained when the IGRA- healthy controls were removed from the analysis, with the MLR higher in IGRA+ subgroup B (median = 0.52) compared to subgroup A (median = 0.35, p = 0.04) (Fig. 4A).

Using a second-degree polynomial model, the MLR was found to change over the time-course of the study period in IGRA+ subgroup B, and was close to the threshold of significance (linear term p = 0.07, quadratic term p = 0.06). This was not observed in IGRA+ subgroup A (linear term p = 0.6, quadratic term p = 0.8) (Fig. 4B and C).

The relative abundances of other cell types including total monocytes, total lymphocytes, total CD4^+^ T cells and neutrophils were also observed to change with time in IGRA+ subgroup B and not subgroup A (Supplementary Fig. 4).

## 4 Discussion

This analysis has demonstrated that IGRA-positive (IGRA+) participants could be stratified according to their whole blood transcriptome into two distinct populations, one of which clustered with IGRA-negative, tuberculosis (TB)-unexposed controls. This separation was not clearly discernible when the transcriptomes of participants were evaluated at baseline in unstimulated whole blood, but rather was unmasked by TB-specific peptide stimulation after 14 days of TB preventive therapy (PT).

We hypothesised that PT would mediate mycobacterial death in participants for whom IGRA positivity was attributable to ongoing viable *Mycobacterium tuberculosis* (*Mtb*) infection and that the resulting immunological response, detected as a whole blood transcriptomic readout, would differentiate such individuals from a group of IGRA+ participants in whom PT would have no anti-mycobacterial effect due to the absence of viable *Mtb*. Our agnostic clustering approach clustered all four IGRA-negative healthy controls with a subgroup of IGRA+s (IGRA+ A), which is strongly suggestive that if indeed these clusters do define *Mtb* viability status then the true latent tuberculosis infection (LTBI) participants lie within the other subgroup (IGRA+ B). The genes differentially expressed between the two clusters through PT were predominantly involved in the immune system, particularly related to intracellular infection, inflammation, chemotaxis and cytokine signalling, indicating a biologically plausible specific response in the IGRA+ B subgroup.

Alternative explanations for the clear separation of these two groups were considered. Rifampicin has important antimicrobial effects against gram-positive organisms and can eliminate upper respiratory tract carriage of gram-negative organisms such as *Neisseria meningitidis* and *Haemophilus influenzae* within 2–4 days. The inclusion of rifampicin/isoniazid treated, IGRA-negative control participants was an attempt to capture and isolate any such non-mycobactericidal effect. In the absence of microbiological sampling and/or microbiome analysis we cannot entirely exclude the possibility that the separation of the groups is attributable to an effect completely unrelated to *Mtb* infection; however two factors which weigh against this alternative explanation are the low prevalence of *N. meningitidis* and *H. influenzae* carriage in this population (<10% combined) and the identification amongst the differentially expressed genes of several genes known to be associated with *Mtb* response pathways. The changes through PT overlapped with reported changes in blood transcriptome during treatment of active TB cases [25,26] and during Isoniazid PT [27]. The monocyte-to-lymphocyte ratio transiently increased only in the IGRA+ B subgroup: this ratio has been linked with TB disease susceptibility and blood transcriptomes [28]. The prevalence of carriage of non-tuberculous mycobacteria in this London-resident population would also be expected to be very low. We considered the possibility that our observations could reflect differences in drug metabolism. Rifampicin induces gene expression changes in hepatocytes [29,30], but after reviewing this literature and publicly available RNASeq data (Gene Expression Omnibus, Series GEO139896) [29] we found no evidence for this (data not shown). This could also be attributed to the fact that our study focused on peripheral blood-associated gene expression changes as compared to the liver-derived hepatocytes described in these previously reported studies. To further investigate any non-specific effects of Rifampicin and Isoniazid, we also compared gene expression at visit 2 versus visit 1 in the healthy controls, using a paired t-test in limma, and found no significantly differentially expressed genes. Finally, we were concerned to exclude all possible artefactual explanations related to sample handling and found no effect association with study site, time to sample processing, study personnel or date of enrolment.

We contend that interferon gamma release assays (IGRA) and tuberculin skin tests (TST) are mis-represented as tests for LTBI, a term which infers viability of *Mtb* with potential to cause future reactivation disease. We believe that the observation that 90% of individuals with positive testing by IGRA/TST do not develop TB disease is more likely to reflect low frequency of persistent viable (“reactivate-able”) infection than low frequency of breakout of *Mtb* replication from long-term immunological control. The empirical evidence that we present in support of this contention is consistent with recent re-evaluations of epidemiological data which suggest that (1) duration of *Mtb* infection viability is likely to be much shorter than previously believed [31] and that (2) reactivation rates in IGRA or TST positive individuals unprotected by PT undergoing immunosuppressive therapy are much lower than would be expected if such testing represented infection truly capable of reactivation [32]. Emerging mathematical modelling outputs add weight to this paradigm shift, suggesting that a significant proportion of *Mtb*-infected individuals achieve self-clearance, leaving a much smaller population with persisting viable *Mtb* infection than previously assumed [33]. Finally, a precedent for lasting anti-mycobacterial immunological reactivity in the absence of bacterial viability already exists in the form of erythema nodosum leprosum, type II reactions to persistent *M. leprae* antigens which are known to occur years after mycobacterial cure.

These blood transcriptional responses to PT suggest that around one third of our IGRA+ study participants had true (viable) LTBI. This study was performed in TB contacts with recent exposure, who are an IGRA+ population at high risk of progression. The proportion with viable infection is predicted to be lower with increasing remoteness in time since exposure, for example in migrants now resident in low-incidence countries [31]. The implications for national and global estimates of LTBI prevalence that rely upon IGRA/TST data are clear and suggest a large overestimation of the size of the global reservoir of potentially reactivatable latent infection; we contend that such data should in future be presented as prevalence of tuberculin sensitivity and that the term LTBI should be used more judiciously. Since all incident reactivation arises from the true LTBI pool, the incidence rate in this subgroup of all IGRA positives will be considerably higher than, for example, the 0.6 per 100 person-years seen in the placebo arm of a recent vaccine trial [34]. The development of tools and strategies to readily identify this true LTBI subgroup would facilitate more efficient targeting of interventions to interrupt reactivation and would accelerate evaluation of novel interventions because the sample size required for future vaccine trials and trials of preventive therapy would be considerably reduced. Evaluations of risk factors associated with infection, premised on the use of IGRA/TST to define infection, have likely been using a very imperfect endpoint with the associated high likelihood of misclassification error.

The temporal dynamics of the transcriptomic changes are such that evidence of a response can be detected as early as 2 weeks into PT. This raises the possibility of a ‘treat and test’ approach to PT wherein the absence of a specific change in a biomarker (or biomarker profile) at an early time point, say 2 weeks into treatment, could be interpreted as an indication that further treatment will have no effect and can then be discontinued. Recent TB host gene expression studies have shown that biomarker signatures can be shrunk to small sets with the potential to be implemented as diagnostic or prognostic tests in the field [[35], [36], [37]].

This is the first study to look at longitudinal transcriptomic responses in the blood of IGRA+ individuals post-stimulation during the course of PT. Despite its novelty and strengths, it has a relatively modest number of participants. Sequential transcriptomic and cell count differential testing on a larger study population in which defined secondary cases are identified, with a variety of exposure histories and diverse PT regimens (including those under investigation for multidrug-resistant LTBI) will help to elucidate the array of responses encountered. The hunt for predictors of future disease amongst TB- exposed individuals has previously been directed towards identification of biomarkers indicating increased risk, an approach that risks dismissal of future changes in the host environment which it might not be possible to anticipate (e.g. transplant immunosuppression). By removing from the pool of *Mtb*-sensitised participants (IGRA+ or TST+) a significant proportion for whom reactivation is biologically impossible (because no viable *Mtb* infection remains), the scale of the prevention challenge is drastically reduced and a more efficient, targeted and nuanced approach can be considered.

Important implications of a test that can distinguish IGRA+ or TST+ *Mtb-*sensitised individuals at zero risk of progression/reactivation include drastic reevaluation of the global burden of LTBI, stratification of preventive therapy and post-exposure vaccine efficacy, higher resolution targeting of LTBI preventive therapy, potential use as a biomarker for efficacy evaluation of novel PT regimens for drug-susceptible and drug-resistant-TB, and PT test of cure.

## 5 Conclusion

Individuals with immunological memory of a prior encounter with *Mtb* (commonly referred to as LTBI) who are treated with PT demonstrate two different phenotypes of transcriptomic response. We propose that the clear responders are those who had truly viable latent *Mtb* infection, and that the minimal responders, in common with the IGRA-negative, previously unexposed healthy controls, had no viable *Mtb* organisms and were therefore not truly latently TB infected.

## Author contributions

Claire Broderick: Conceptualisation, Methodology, Investigation, Formal analysis, Data curation, Writing- original draft, Visualisation, Project administration, Funding acquisition.

Jacqueline Cliff: Methodology, Investigation, Formal analysis, Data curation, Resources, Writing- original draft, Funding acquisition.

Ji-Sook Lee: Investigation, Data curation, Resources.

Myrsini Kaforou: Methodology, Formal analysis, Writing-review and editing, Visualisation.

David Moore: Conceptualisation, Methodology, Formal analysis, Writing- original draft, Supervision, Funding acquisition.

## Appendix A Supplementary data

Supplementary file 1 Significantly differentially expressed (SDE) transcripts IGRA+ *vs* IGRA- in TB1 tubes at Visit (V) 1 and V2 and in TB2 tubes at V1 and V2. Supplementary file 1

Supplementary file 2 SDE transcripts TB1 *vs* negative tube, TB2 *vs* negative tube at V1, in IGRA+. Supplementary file 2

Supplementary file 3 SDE transcripts TB1 *vs* negative tube, TB2 *vs* negative tube at V1, in IGRA-. Supplementary file 3

Supplementary file 4 474 most variable transcripts (TB2-stimulated samples). Supplementary file 4

Supplementary file 5 MaSigPro results: transcripts SDE though time, Cluster 1 *vs* Cluster 2. Supplementary file 5

Supplementary file 6 Functional classification of transcripts differentially expressed between Cluster 1 and Cluster 2. Supplementary file 6

Supplementary file 7 Results of biological pathways analysis using Reactome pathway knowledgebase. Supplementary file 7

Supplementary figure 1 **Principle component analyses of the initial gene expression sets.** Plots showing dimensions 1 and 2 of the principle component analyses of the PAXgene samples (A) and the stimulated samples (B) before ComBat correction. In the stimulated samples, a healthy control (HC52) was an outlier in dimensions 1 and 2 (circled) and this persisted after batch correction (not shown), so HC52 was excluded from the subsequent analyses. Supplementary figure 1

Supplementary figure 2 **Principle component analyses of the gene expression sets before and after ComBat.** Gene expression data from 18 IGRA+ and 4 IGRA- participants were included in the final analyses. Batch correction was performed with ComBat. Plots showing dimensions 1 and 2 of the principle component analyses of the PAXgene tube samples before (A) and after ComBat (B); all stimulated samples (TB1, TB2 and Negative) before (C, D) and after ComBat (E, F) with C and E showing batch differentiation and D and F showing tube differentiation; TB1 samples before (G) and after Combat (H); TB2 samples before (I) and after Combat (J). Batch, visit, IGRA status and QuantiFERON TB Gold plus tube are provided. Supplementary figure 2

Supplementary figure 3 **Volcano plots showing genes significantly differentially expressed between stimulated (QuantiFERON Gold Plus TB1 and TB2 tubes) and unstimulated (QuantiFERON Gold Plus negative tubes) blood samples**. Plots are shown for IGRA+ subjects, comparing TB1 *vs.* negative tube samples (A), and TB2 *vs*. negative tube samples (B) at visit 1. Also shown are plots for IGRA- subjects, comparing TB1 *vs*. negative tube samples (C), and TB2 *vs*. negative tube samples (D) at visit 1. Genes overexpressed in stimulated blood with log2Foldchange (LFC) >1 and BH adjusted p value < 0.05 are shown in red. Genes underexpressed in stimulated blood with LFC <-1 and BH adjusted p value < .05 are shown in blue. Genes with LFC >2.7 and < -1.7 are annotated with their gene symbols. Dotted line denotes the significance cut-off (BH adjusted p value < 0.05). Supplementary figure 3

Supplementary figure 4 **Longitudinal changes in cellular populations through preventive therapy in IGRA+ subgroups A and B.** Cibersortx was used to estimate the abundance of different cell types in the TB2-stimulated whole blood samples at each visit. Scatterplots showing the change cellular fractions over the time-course of the study period in IGRA+ subgroups A and B for Total monocyte fraction (A), Total lymphocyte fraction (B), Total CD4+ T cell fraction (C), Neutrophil fraction (D). Visit 1 is 0 weeks, Visit 2 is 2 weeks and Visit 3 is 13 weeks, with 90% confidence intervals shown. Supplementary figure 4
