# Supplementary figures and images for: Host transcriptional response to TB preventive therapy differentiates two sub-groups of IGRA-positive individuals
Source: Tuberculosis (Edinb). 2021 Mar;127:102033. doi: 10.1016/j.tube.2020.102033 (PMC7985621; doi:10.1016/j.tube.2020.102033)

**A**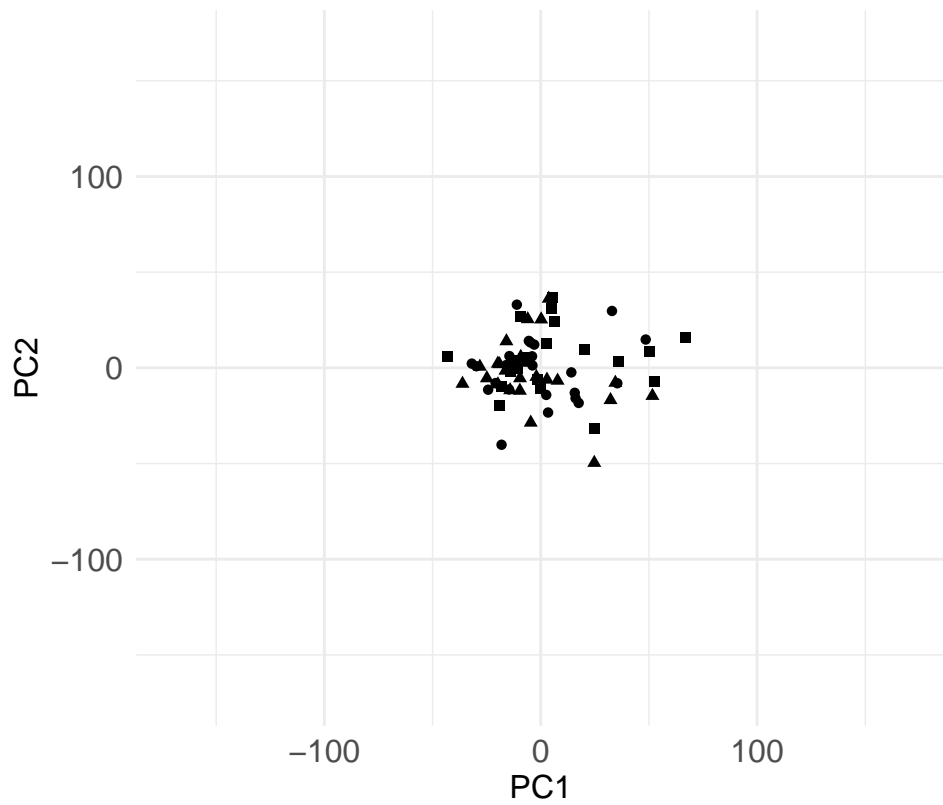**B**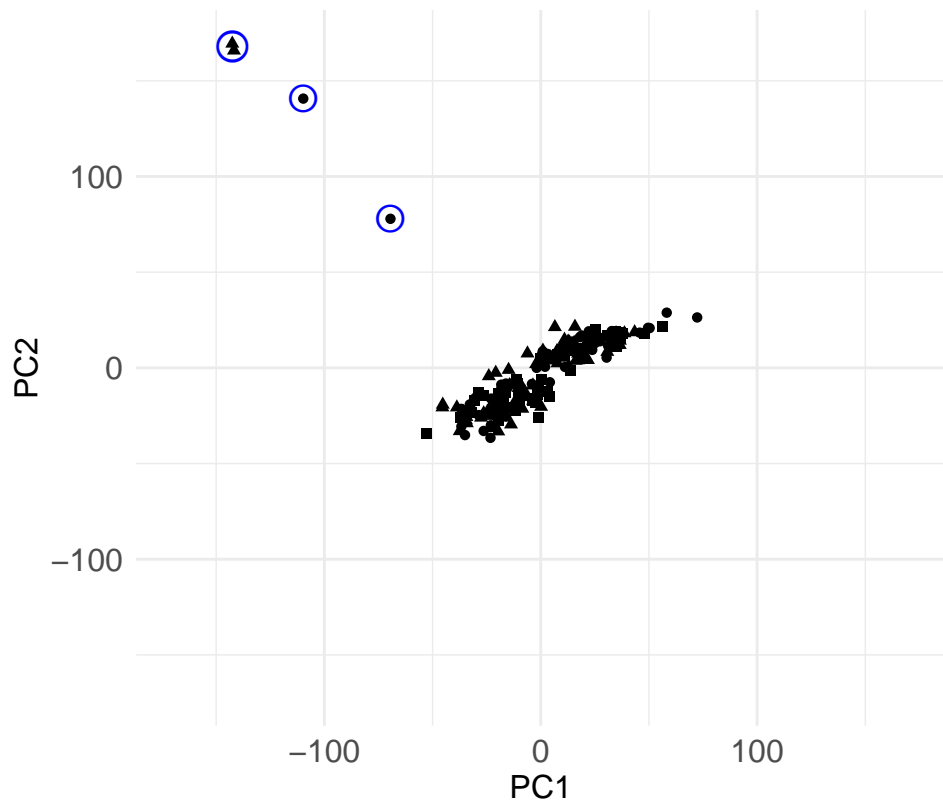

Visit • V1 ▲ V2 ■ V3

Supplement: Supplementary figure 1 — Principle component analyses of the initial gene expression sets. Plots showing dimensions 1 and 2 of the principle component analyses of the PAXgene samples (A) and the stimulated samples (B) before ComBat correction. In the stimulated samples, a healthy control (HC52) was an outlier in dimensions 1 and 2 (circled) and this persisted after batch correction (not shown), so HC52 was excluded from the subsequent analyses. [file mmc8.pdf]

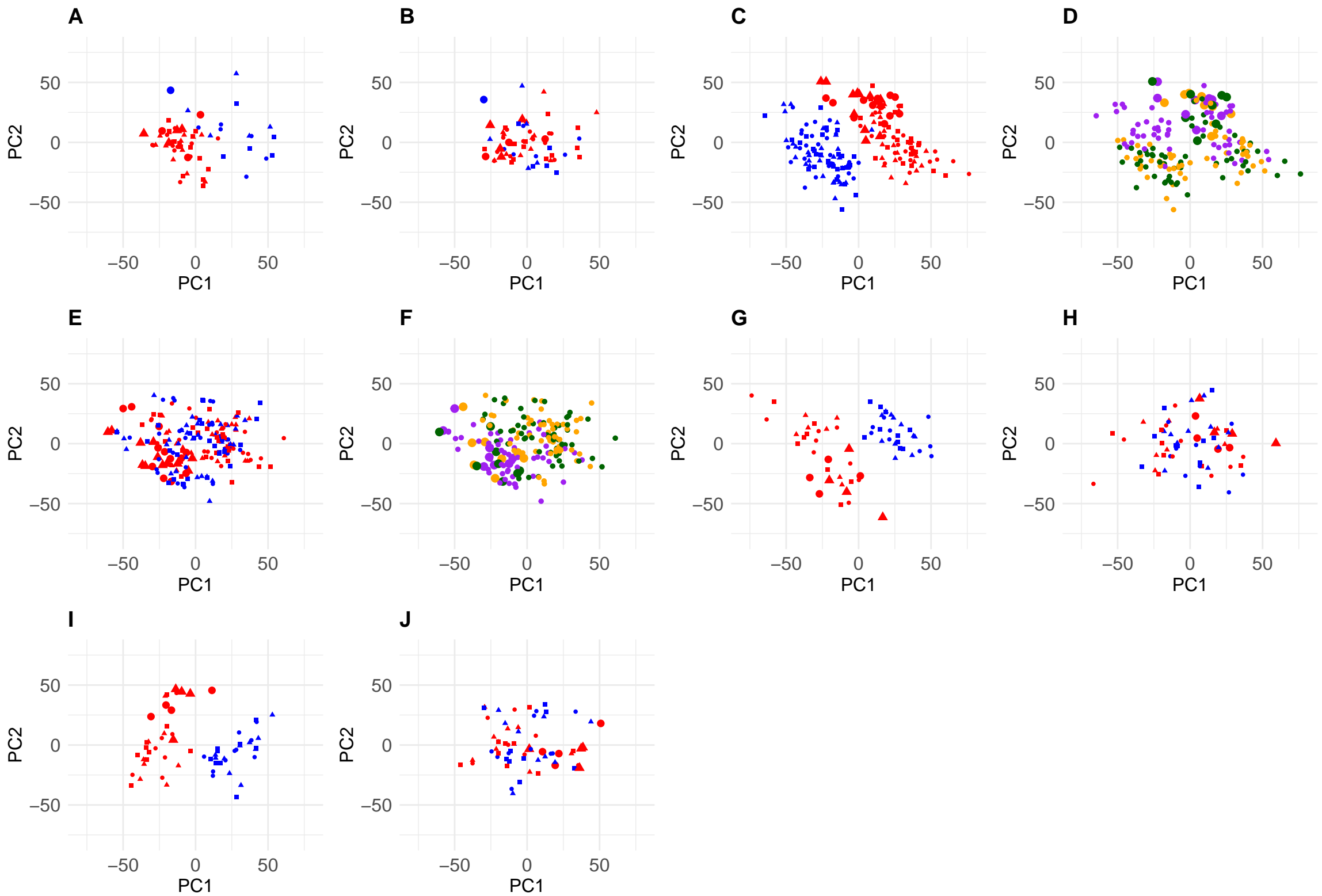

Visit • V1 ▲ V2 ■ V3 Batch • A • B Condition ● IGRA- ● IGRA+

Tube • Neg • TB1 • TB2

Supplement: Supplementary figure 2 — Principle component analyses of the gene expression sets before and after ComBat. Gene expression data from 18 IGRA+ and 4 IGRA- participants were included in the final analyses. Batch correction was performed with ComBat. Plots showing dimensions 1 and 2 of the principle component analyses of the PAXgene tube samples before (A) and after ComBat (B); all stimulated samples (TB1, TB2 and Negative) before (C, D) and after ComBat (E, F) with C and E showing batch differentiation and D and F showing tube differentiation; TB1 samples before (G) and after Combat (H); TB2 samples before (I) and after Combat (J). Batch, visit, IGRA status and QuantiFERON TB Gold plus tube are provided. [file mmc9.pdf]

**A**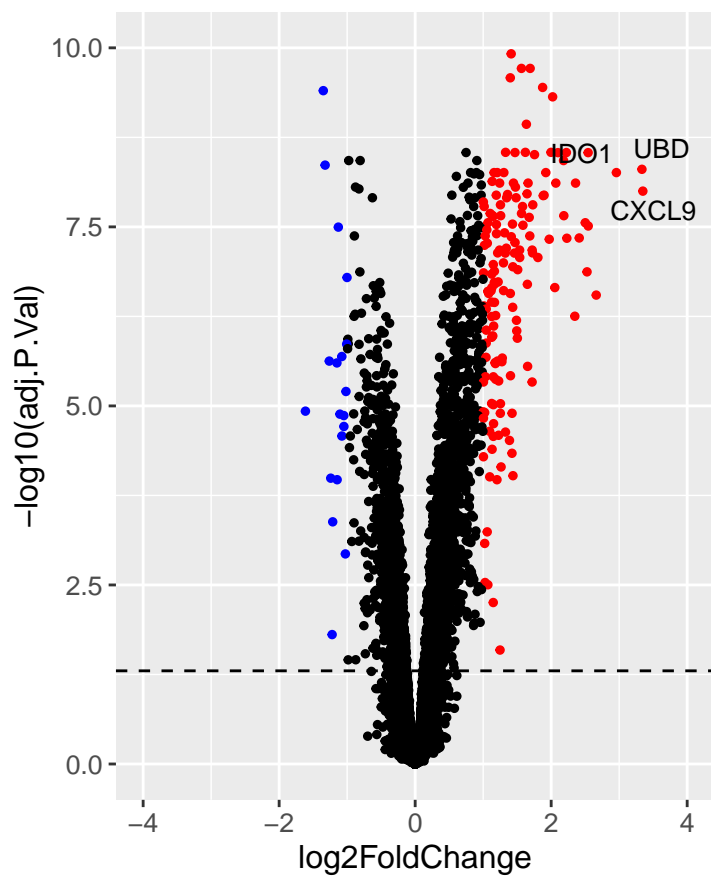**B**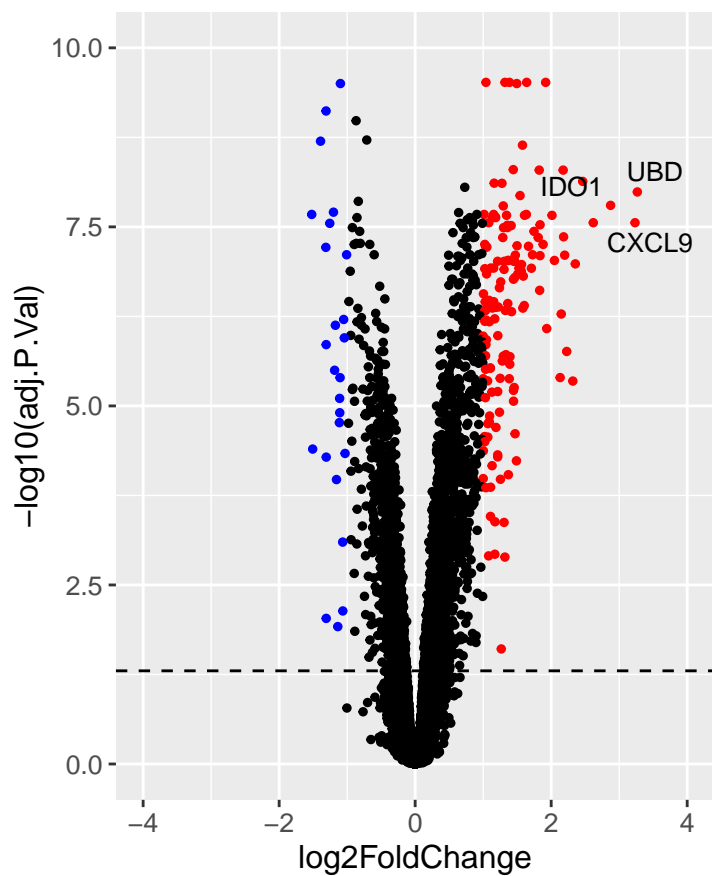**C**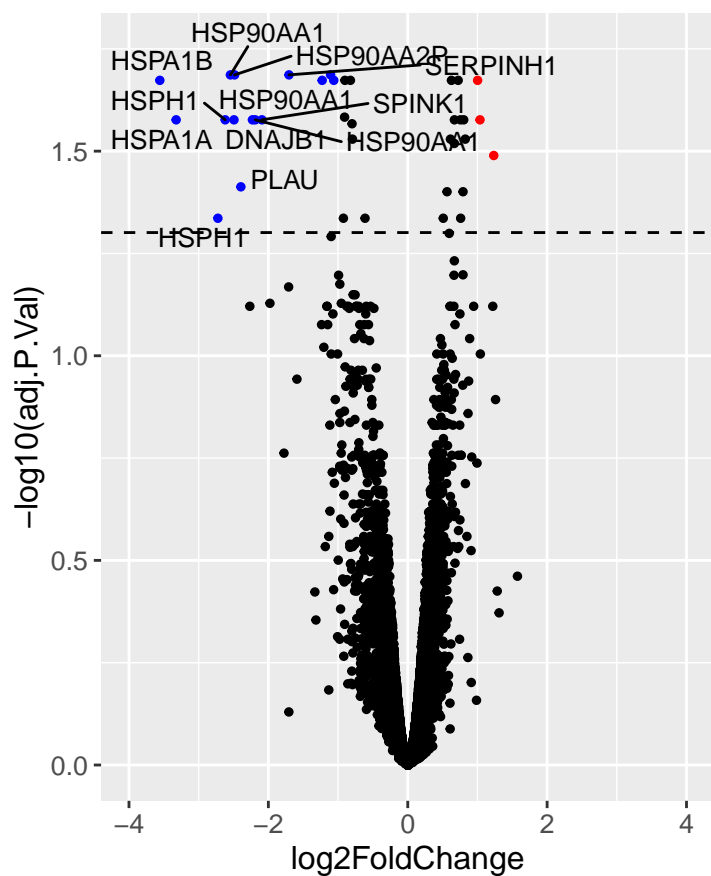**D**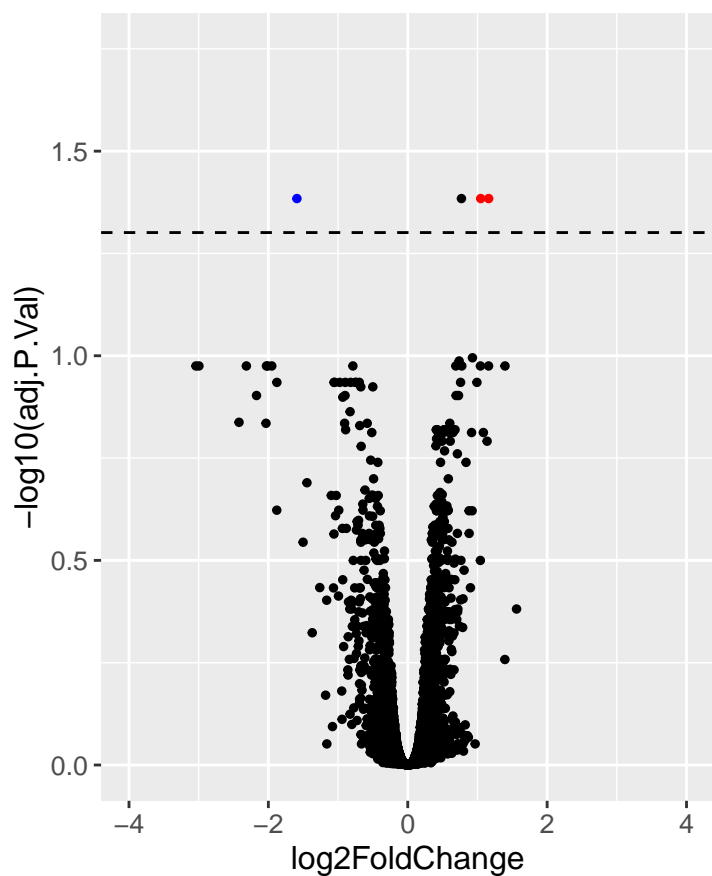

Supplement: Supplementary figure 3 — Volcano plots showing genes significantly differentially expressed between stimulated (QuantiFERON Gold Plus TB1 and TB2 tubes) and unstimulated (QuantiFERON Gold Plus negative tubes) blood samples. Plots are shown for IGRA+ subjects, comparing TB1 vs. negative tube samples (A), and TB2 vs. negative tube samples (B) at visit 1. Also shown are plots for IGRA- subjects, comparing TB1 vs. negative tube samples (C), and TB2 vs. negative tube samples (D) at visit 1. Genes overexpressed in stimulated blood with log2Foldchange (LFC) >1 and BH adjusted p value < 0.05 are shown in red. Genes underexpressed in stimulated blood with LFC <-1 and BH adjusted p value < .05 are shown in blue. Genes with LFC >2.7 and < -1.7 are annotated with their gene symbols. Dotted line denotes the significance cut-off (BH adjusted p value < 0.05). [file mmc10.pdf]

**A. Subgroup A**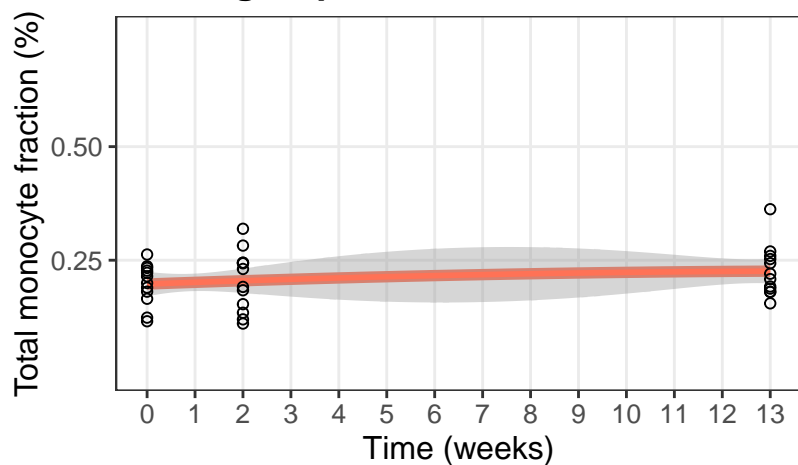**B. Subgroup A**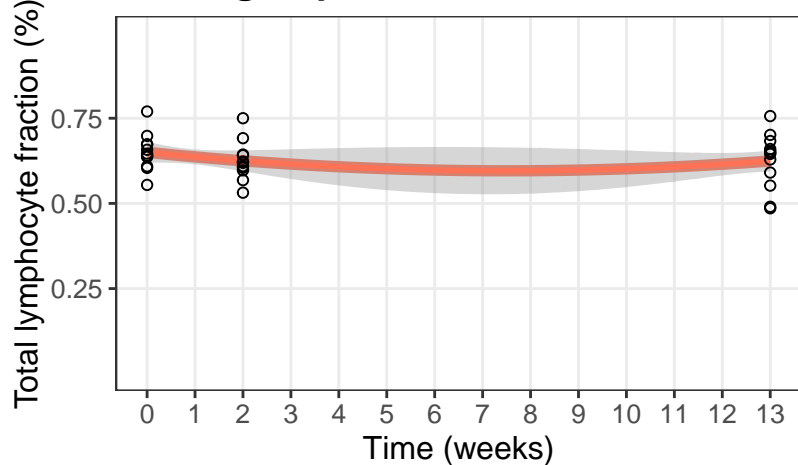**Subgroup B**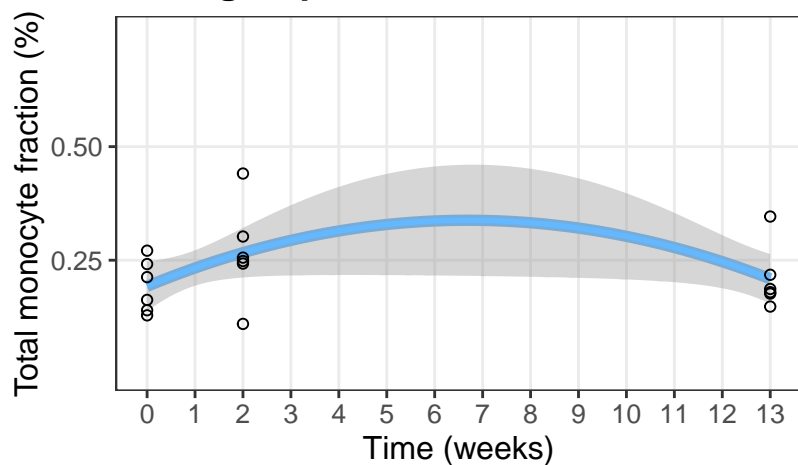**Subgroup B**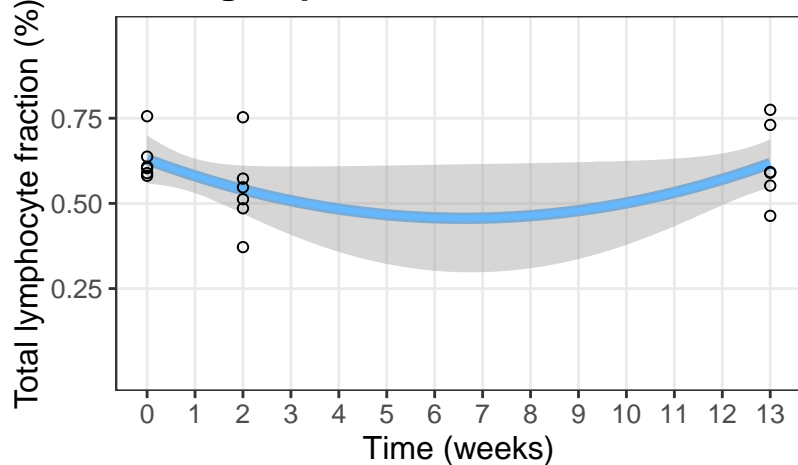**C. Subgroup A**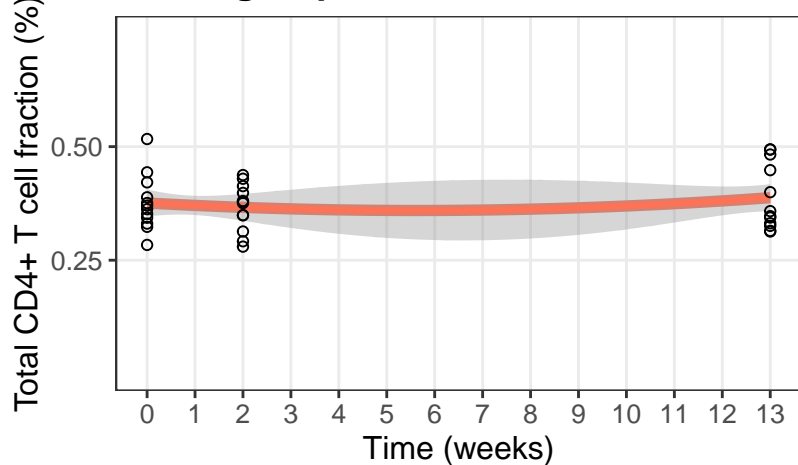**D. Subgroup A**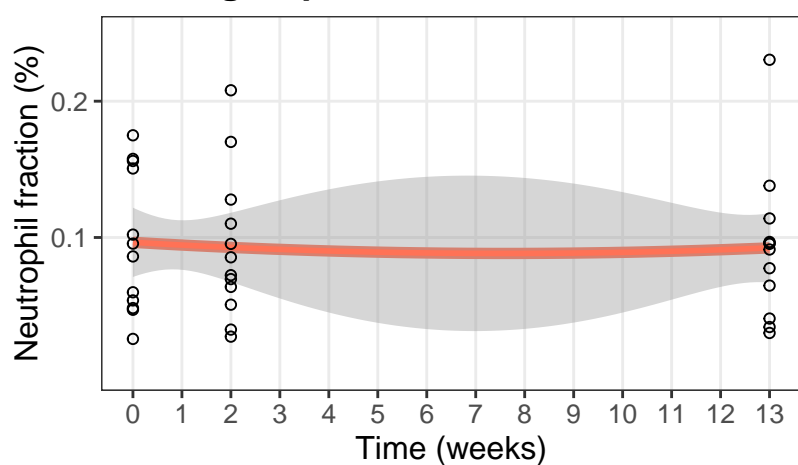**Subgroup B**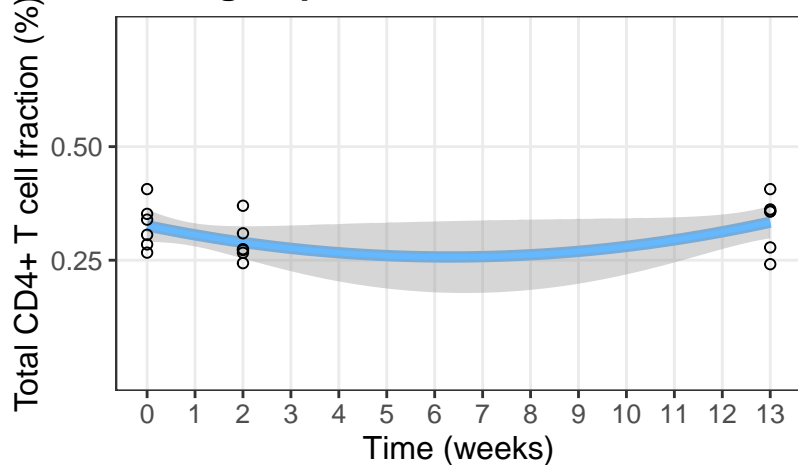**Subgroup B**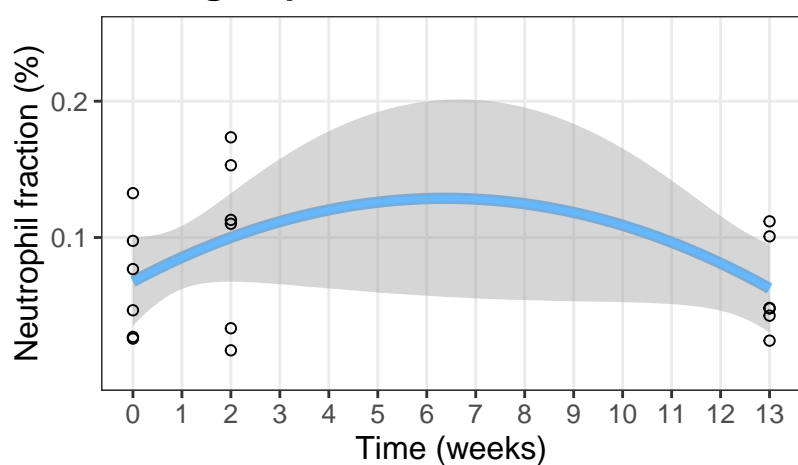

Supplement: Supplementary figure 4 — Longitudinal changes in cellular populations through preventive therapy in IGRA+ subgroups A and B. Cibersortx was used to estimate the abundance of different cell types in the TB2-stimulated whole blood samples at each visit. Scatterplots showing the change cellular fractions over the time-course of the study period in IGRA+ subgroups A and B for Total monocyte fraction (A), Total lymphocyte fraction (B), Total CD4+ T cell fraction (C), Neutrophil fraction (D). Visit 1 is 0 weeks, Visit 2 is 2 weeks and Visit 3 is 13 weeks, with 90% confidence intervals shown. [file mmc11.pdf]
